# Are preterm birth and intra-uterine growth restriction more common in Western Australian children of immigrant backgrounds? A population based data linkage study

**DOI:** 10.1186/s12884-019-2437-x

**Published:** 2019-08-09

**Authors:** Ifrah Abdullahi, Kingsley Wong, Emma Glasson, Raewyn Mutch, Nicholas de Klerk, Jenny Downs, Sarah Cherian, Helen Leonard

**Affiliations:** 1 Telethon Kids Institute, The University of Western Australia, PO Box 855, West Perth, WA 6872 Australia; 2 School of Paediatrics and Child Health, The University of Western Australia, Perth, WA Australia; 3 Department of General Paediatrics, Perth Children’s Hospital, Perth, WA Australia; 4 School of Physiotherapy and Exercise Science, Curtin University, Perth, WA Australia

**Keywords:** Preterm birth, Growth restriction, Apgar score, Immigrant mothers, Data linkage

## Abstract

**Background:**

To compare the prevalence of preterm birth, post term birth, intra-uterine growth restriction and distribution of Apgar scores in offspring of foreign-born women in Western Australia with that of their Australian-born non-Indigenous and Indigenous counterparts.

**Methods:**

A population-based linked data study, involving 767,623 singleton births in Western Australia between 1980 and 2010 was undertaken. Neonatal outcomes included preterm birth, post term births, intra-uterine growth restriction (assessed using the proportion of optimal birth weight) and low Apgar scores. These were compared amongst foreign-born women from low, lower-middle, upper middle and high income countries and Australian-born non-Indigenous and Indigenous women over two different time periods using multinomial logistic regression adjusted for covariates.

**Results:**

Compared with Australian born non-Indigenous women, foreign-born women from low income countries were at some increased risk of extreme preterm (aRRR 1.59, 95% CI 0.87, 2.89) and very early preterm (aRRR 1.63, 95% CI 0.92, 2.89) births during the period from 1980 to 1996. During the period from 1997 to 2010 they were also at some risk of extreme preterm (aRRR 1.42, 95% CI 0.98, 2.04) very early preterm (aRRR 1.34, 95% CI 1.11, 1.62) and post term birth (aRRR 1.93, 95% CI 0.99, 3.78). During this second time period, other adverse outcomes for children of foreign-born women from low income and middle income countries included increases in severe (aRRR 1.69, 95% CI 1.30, 2.20; aRRR 1.72, 95% CI 1.53, 1.93), moderate (aRRR 1.54, 95% CI 1.32, 1.81; aRRR 1.59, 95% CI 1.48, 1.70) and mild (aRRR 1.28, 95% CI 1.14, 1.43; aRRR 1.31, 95% CI 1.25, 1.38) IUGR compared to children of Australian-born non-Indigenous mothers. Uniformly higher risks of adverse outcomes were also demonstrated for infants of Indigenous mothers.

**Conclusions:**

Our findings illustrate the vulnerabilities of children born to foreign women from low and middle-income countries. The need for exploratory research examining mechanisms contributing to poorer birth outcomes following resettlement in a developed nation is highlighted. There is also a need to develop targeted interventions to improve outcomes for these women and their families.

**Electronic supplementary material:**

The online version of this article (10.1186/s12884-019-2437-x) contains supplementary material, which is available to authorized users.

## Background

Relationships between adverse pregnancy and birth outcomes such as preterm birth, intra-uterine growth restriction (IUGR) and low Apgar scores and increased infant morbidity and mortality are well-recognised [1–8]. There is also a further body of research evidence linking abnormal pregnancy and birth outcomes to the development of neurodevelopmental disorders and child disability [2, 5, 8, 9]. For instance, associations between the presence of IUGR and subsequent intellectual disability (ID) have been demonstrated in Western Australian (WA) population-based research while premature children with extremely low and very low birth weights also have an increased risk of autism spectrum disorders (ASD) [8–10].

Several studies have found that foreign-born women living in developed countries are at increased risk of adverse obstetric and perinatal outcomes including preterm delivery, low birth weight, IUGR, Caesarean section, postpartum haemorrhage and neonatal mortality and infection [11–17]. Obstetric profiles, including maternal medical conditions and pregnancy complications, of foreign-born women of culturally and linguistically diverse (CALD) backgrounds have also been compared with those of Australian-born women [11, 18, 19] using population-based data linkage. In these studies foreign-born women, especially those from Sub-Saharan Africa, Southeast Asia and Southern and Central Asia, were more likely to undergo emergency Caesarean section, often because of failure of labour to progress and fetal distress [18, 19]. They were also at increased risk of gestational diabetes, pre-labour rupture of membranes, perineal laceration and post-partum haemorrhage [11]. Foreign-born mothers at risk have often been categorised by region, or for an individual country of birth the numbers may have been too small to establish any meaningful risk profiles [13, 16]. Findings have been variable [20], with only some identifying a relationship with preterm birth [14]. The literature demonstrates the variability in the accuracy of the measurement of fetal growth in children of immigrant backgrounds [15, 16], and suggests that the use of fetal and newborn charts as single diagnostic tools (birthweight and gestational age charts) in determining perinatal outcomes may be debatable [21, 22].

Australia is a multiethnic society with just over a quarter of citizens (28%) born overseas [23], including a smaller subset of refugees. Overseas migration and resettlement is predominantly from Europe with smaller numbers from North Africa, Middle East, South East Asia and Sub-Saharan Africa [20, 24, 25]. Although research to date documenting pregnancy and birth outcomes among these foreign-born women suggests that their obstetric profiles may differ from those of Australian-born women, the body of evidence remains limited. The aim of this current study was to compare the prevalence of preterm and post term births, intra-uterine growth restriction and distribution of Apgar scores in offspring of foreign-born women in WA with that of their Australian-born Caucasian and Indigenous counterparts.

## Methods

### Study population and data sources

A retrospective cohort study of children born in WA (1980 to 2010) was conducted using de-identified linked population-based data from the WA Midwives Notification System (MNS) and the WA Birth Register. The MNS is a statutory database of pregnancy, birth and neonatal information collected by attending midwives, on all births of at least 20 weeks’ gestation or at least 400 g in weight (if unknown gestational age) and includes maternal and infant characteristics and infant outcomes. For this study, multiple births were excluded because they are associated both with poorer birth outcomes and country of birth [26]. The WA Birth Register contains all registered births and includes maternal, paternal and infant demographic characteristics. The birth cohort was subdivided into two time periods (P1; 1980 to 1996 and P2; 1997 to 2010), to allow for adjustment for smoking which was only collected after 1996, and because of changes in migration patterns over time.

### Maternal variables

Maternal variables collected were maternal age (maternal age in years at the time of delivery), marital status (never married, widowed, divorced, separated, married and unknown), parity (the number of live born and stillbirths a woman has had leading up to this particular pregnancy), and smoking (a binary variable only collected in the MNS from September 1997).

Six categories were created using the maternal country of birth (MCB) variable, available from the Birth Register: 1) Australian-born mothers of non-Indigenous backgrounds (ANI), 2) Australian-born mothers of Indigenous background (AI), 3) foreign-born mothers from low-income countries (FB-LIC), 4) foreign-born mothers from lower-middle-income countries (FB-LMIC), 5) foreign-born mothers from upper-middle-income countries (FB-UMIC), and 6) foreign-born mothers from high-income countries (FB-HIC). Countries’ income groups were determined using the United Nations, World Health Organization and World Bank country listings [27–31] and income groups were categorized according to gross national income per capita in 2017. Income brackets and thresholds for gross national income per capita were < US$1005 for low-income, US$1006–3955 for lower-middle-income, US$3956–12,235 for upper-middle-income, and > US$12,235 for high-income [32]. Low and middle-income country groups as per the World Bank categorization are listed in Additional file 1: Table S1; high-income economies included the major migrant groups to Australia such as those from the UK and New Zealand (Additional file 2: Table S2).

The Australian Bureau of Statistics produces the Socio-Economic Indexes for Areas (SEIFA) score, which ranks areas in Australia according to relative socio-economic advantage and disadvantage from information collected during the Australian Census [33]. We specifically used the Index of Relative Socio-economic Advantage and Disadvantage (IRSAD), an index that summarises the information about the economic and social conditions of people and households within a census district [33]. This index includes measures of relative advantage and disadvantage; with scores of 1 representing the lowest score (relative lack of disadvantage) on the index and 10 the highest score of the index (greatest disadvantage). IRSAD scores were grouped into six categories: advantaged (90% of the IRSAD score), least disadvantaged (76–90% of the IRSAD score),less disadvantaged (51–75% of the IRSAD score), little disadvantage (25–50% of the IRSAD score), more disadvantage (10–25% of the IRSAD score), and the most disadvantaged (lowest 10%) areas of WA [33]. We used the IRSAD score for the census district where the mother was living at the time of birth [33].

### Child variables

Infant variables were gender, gestational age, proportion of optimal birth weight (POBW) and Apgar score at 5 min. The adverse fetal outcomes were: (i) preterm birth, (ii) IUGR and (iii) low Apgar score. Preterm birth was determined using a final gestational age variable that was categorized into seven groups of gestation: extreme preterm (< 28 weeks), very early preterm (28–31 weeks), early preterm (32–33 weeks), late preterm (34–36 weeks), early term (37–38 weeks), full term (39–41 weeks), and post term (> 42 weeks). Any pre-term was defined as < 37 weeks and the category of full term birth (39–41 weeks) was used as the reference group for the multinomial regression data analysis. IUGR was determined using the POBW, which is the ratio of observed birth weight to optimal birth weight, and is a variable that is calculated and provided to researchers by the data custodians in WA.

The algorithm from which the optimal birth weight is calculated was developed from a regression model accounting for non-pathological causes of fetal size (gestational age, maternal height, maternal parity and infant sex) in a total population of singleton births without any recorded risk factors for intrauterine growth restriction such as maternal smoking [34, 35]. The percentage was categorized into seven groups: < 75%, 75–84%, 85–94%, 95–104%, 105–114%, 115–124, and > 125% with < 75% considered as severe, 75–84% as moderate and 85–94% as mild growth restriction. The middle group, 95–104% was used as the reference group in the multinomial regression data analysis [35]. The reference group comprised almost one third (32.1%) of the total population; POBW less than 85% was equivalent to approximately less than the 10th percentile of optimal birth weight [36] and POBW greater than 115% was equivalent to greater than the 90th percentile of optimal birth weight. Apgar score was determined using the Apgar variable at 5 min and the categories were defined as normal (7 to 10), fairly low (4 to 6) and critically low (3 or less), with the normal group being used as the reference group in the analysis. Apgar score at 1 min was excluded from analyses due to inadequate reporting or missing data.

### Analyses

Descriptive statistics were used to summarise the characteristics of the study participants. Multinomial logistic regression models were used to estimate the risk of pre/post term birth relative to full term birth among MCB groups compared to Australian-born women of non-Indigenous background, after adjusting for potential confounders including maternal age, SEIFA score, parity, birth year and child’s sex. The model was also used for assessing the risk of suboptimal (POBW less than 95% or more than 104%) relative to optimal (POBW between 95 and 104%) birth weight and risk of low relative to normal Apgar score. Both crude and adjusted relative risk ratios, and their 95% confidence interval values were reported. Missing data were considered missing at random and complete case analysis was used. Since smoking data were only available from September 1997, we conducted regression analysis separately for the two-time periods: 1980–1997 (without adjusting for smoking) and 1997–2010 (with adjusting for smoking). All analyses were performed using STATA version 14 (StataCorp LP, College Station, TX, USA).

## Results

**Fig. 1:**
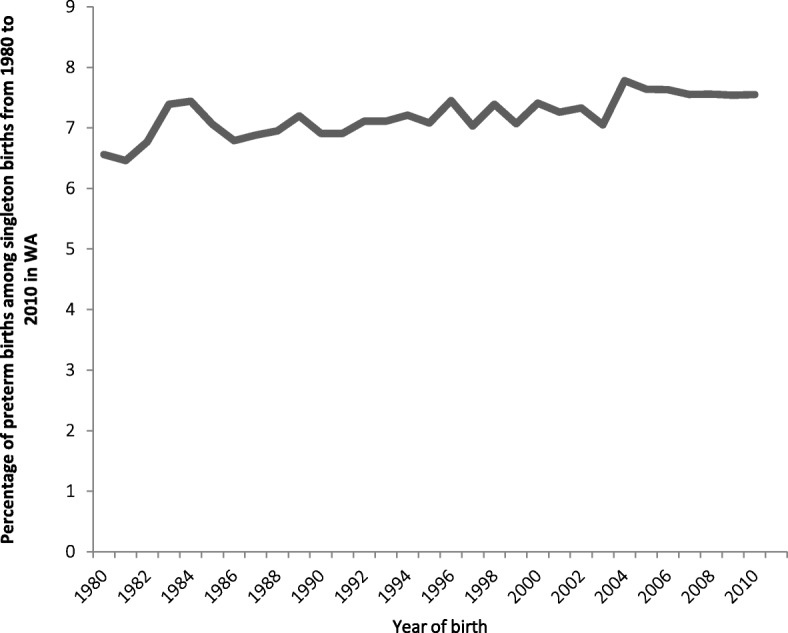
Prevalence of preterm birth in singleton births from 1980 to 2010 in WA

**Fig. 2:**
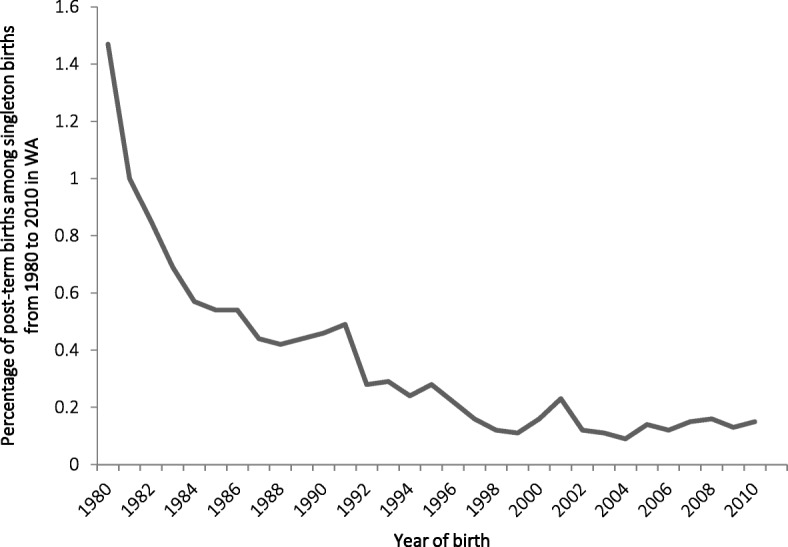
Prevalence of post-term births in singleton births from 1980 to 2010 in WA

For the years 1980 to 2010 our study included 769,695 singleton births with a valid MCB (out of a total 792,373 WA births during that period) of which 66.2% were children of ANI mothers, 19.0% were children of FB-HIC mothers, 5.8% were children of AI mothers, 4.3% were children of FB-LMIC mothers, 4.1% were children of FB-UMIC, and 0.6% were children of FB-LIC. Only 0.1% of our sample population had missing data for MCB. Socio-demographic factors including smoking status are presented in Table 1. AI mothers were more likely to be aged less than 20 years (27.2%) compared to ANI mothers and foreign mothers, whilst FB-LMIC mothers were less likely to be aged less than 20 years of age than ANI mothers (1.9 vs 5.6%). FB-UMIC mothers were more likely to be married (96.0%) followed by FB-LMIC (94.5%), FB-HIC (92.4%) and FB-LIC (91.7%) compared with ANI mothers (90.1%) and AI mothers (62.8%). As shown in Table 1 AI mothers had the lowest (0–2) and highest (3–5) parity scores, whilst FB-LIC mothers had lower parity levels (parity 0–2, 31.8, 29.6 and 17.8% respectively) compared to ANI mothers (parity 0–2, 41.5, 40 and 16.5% respectively). Yet compared to ANI and other foreign-born, FB-LIC mothers had the second highest parity levels (3, 4, and 5) after AI mothers. Only 13.3% of FB-LMIC and 12.9% FB-LIC mothers were categorised as most disadvantaged, in comparison to AI mothers of whom 35.0% were categorised as such. Smoking was least prevalent in foreign-born, particularly FB-LIC (1.9%), FB-LMIC (2.8%), FB-UMIC (5.9%) respectively, compared with mothers in the other groups (AI 50.7% and ANI 18.3%). The prevalence of preterm birth (< 37 weeks) increased from 6.6% in 1980 to 7.6% in 2010 and with an average 7.2% over the whole-time period (Fig. 1), whilst post term births decreased over time (Fig. 2).

**Table 1:** Characteristics of the study population (singleton births from 1980 to 2010 in WA) and their mothers by maternal country of birth group

	ANI (*n* = 509,589)	AI (*n* = 44,820)	FB-LIC (*n* = 4458)	FB-LMIC (*n* = 33,315)	FB-UMIC (*n* = 31,254)	FB-HIC (*n* = 146,259)	Total (*n* = 769,695)
Gender							
Female	248,116 (48.7)	21,868 (48.8)	2175 (48.8)	16,076 (48.3)	15,038 (48.1)	71,344 (48.8)	374,617 (48.7)
Male	261,455 (51.3)	22,943 (51.2)	2282 (51.2)	17,238 (51.7)	16,212 (51.9)	74,909 (51.2)	395,039 (51.3)
Total	509,571 (100)	44,811 (100)	4457 (100)	33,314 (100)	31,250 (100)	146,253 (100)	769,656 (100)
Pearson chi2 (5) 7.1953, *Pr* = 0.207							
Maternal age							
< 20 years	28,763 (5.6)	12,179 (27.2)	118 (2.7)	629 (1.9)	675 (2.2)	4587 (3.1)	46,951 (6.1)
20–24 years	107,752 (21.2)	15,375 (34.3)	777 (17.4)	4831 (14.5)	4331 (13.9)	23,496 (16.1)	156,562 (20.3)
25–29 years	175,640 (34.5)	10,012 (22.3)	1438 (32.3)	11,006 (33.0)	9505 (30.4)	45,334 (31.0)	252,935 (32.9)
30–34 years	137,957 (27.1)	5032 (11.2)	1300 (29.2)	10,661 (32.0)	10,360 (33.2)	46,067 (31.5)	211,377 (27.5)
35–39 years	51,455 (10.1)	1877 (4.2)	682 (15.3)	5155 (15.5)	5276 (16.9)	22,379 (15.3)	86,824 (11.3)
> 39 years	8009 (1.6)	345 (0.8)	143 (3.2)	1033 (3.1)	1106 (3.5)	4395 (3.0)	15,031 (2.0)
Total	509,576 (100)	44,820 (100)	4458 (100)	33,315 (100)	31,253 (100)	146,258 (100)	769,680 (100)
Pearson chi2 (25) 6.0 × 10^+ 4^ *Pr* < 0.001							
Marital status							
Never married	43,661 (8.6)	15,482 (34.5)	239 (5.4)	1318 (4)	878 (2.8)	9110 (6.2)	70,688 (9.2)
Widowed	335 (0.07)	78 (0.2)	10 (0.4)	50 (0.2)	15 (0.05)	88 (0.3)	576 (0.07)
Divorced	1812 (0.4)	95 (0.2)	18 (0.4)	91 (0.3)	85 (0.3)	618 (0.4)	2719 (0.4)
Separated	3620 (0.7)	682 1.5)	84 (1.9)	251 (0.8)	180 (0.6)	1004 (0.7)	5821 (0.8)
Married	459,242 (90.1)	28,167 (62.8)	4089 (91.7)	31,491 (94.5)	29,994 (96.0)	135,159 (92.4)	688,142 (89.4)
Unknown	919 (0.2)	316 (0.7)	18 (0.4)	114 (0.3)	102 (0.3)	280 (0.2)	1749 (0.2)
Total	509,589 (100)	44,820 (100)	4458 (100)	33,315 (100)	31,254 (100)	146,259 (100)	769,695 (100)
Pearson chi2 (25) 4.1 × 10^+ 4^ *Pr* < 0.001							
SEIFA score							
0–10	44,509 (9.9)	12,552 (35)	535 (12.9)	4071 (13.3)	2582 (8.9)	10,895 (8.2)	75,144 (11)
11–25	74,190 (16.5)	8691 (24.3)	762 (18.5)	5000 (16.3)	3933 (13.6)	19,911 (15)	112,487 (16.5)
26–50	116,710 (26)	8,141 (22.7)	1169 (28.4)	7965 (26)	6912 (23.9)	33,416 (25.1)	174,313 (25.6)
51–75	113, 336 (25.2)	4749 (13.3)	868 (21.1)	7292 (23.8)	7535 (26)	34,856 (26.2)	168,636 (24.7)
76–90	63,480 (14.1)	1375 (3.8)	490 (11.9)	3881 (12.7)	4802 (16.6)	21,121 (15.9)	95,149 (13.9)
> 90	37,467 (8.3)	321 (0.9)	295 (7.2)	2450 (8)	3174 (11)	12,901 (9.7)	56,608 (8.3)
Total	449,692 (100)	35,829 (100)	4119 (100)	30,659 (100)	28,938 (100)	133,100 (100)	682,337 (100)
Pearson chi2 (25) 3.1 × 10^+ 4^ *Pr* < 0.001							
Parity							
0	210,158 (41.5)	13,122 (30.7)	1360 (31.8)	13,932 (42.2)	12,725 (41)	57,523 (39.6)	308,820 (40.5)
1	171,955 (40)	10,392 (24.3)	1267 (29.6)	11,375 (34.4)	10,998 (35.4)	50,008 (34.5)	255,995 (33.6)
2	83,577 (16.5)	8097 (19)	763 (17.8)	4999 (15.1)	4908 (15.8)	24,633 (17)	126,977 (16.7)
3	28,546 (5.6)	5585 (11.1)	467 (11)	1830 (5.5)	1611 (5.2)	8978 (6.25)	47,017 (6.2)
4	8980 (1.8)	3466 (8.1)	269 (6.3)	667 (2.0)	573 (2)	2955 (2)	16,910 (2.2)
5 & more	3044 (0.6)	2034 (4.8)	157 (3.7)	249 (0.8)	218 (0.7)	1057 (0.7)	6759 (0.9)
Total	506,260 (100)	42,696 (100)	4283 (100)	33,052 (100)	31,033 (100)	145,154 (100)	762,478 (100)
Pearson chi2 (25) 2.2 × 10^+ 4^ *Pr* < 0.001							
Smoking							
Yes	42,866 (18.3)	10,892 (50.7)	58 (1.9)	507 (2.8)	1034 (5.9)	8543 (15.2)	63,900 (18.2)
No	191,678 (81.7)	10,603 (49.3)	3023 (98.1)	17,660 (97.2)	16,617 (94.4)	47,642 (84.8)	287,223 (81.8)
Total	234,544 (100)	21,495 (100)	3081 (100)	18,167 (100)	17,651 (100)	56,185 (100)	351,123 (100)
Pearson chi2 (25) 2.1 × 10^+ 4^ *Pr* < 0.001

Perinatal outcomes (preterm birth, IUGR and Apgar scores) in relation to maternal country of birth are presented in Tables 2, 3, 4, 5, 6 and 7 respectively.

**Table 2:** Risk of pre/post term relative to full term by maternal country of birth: 1980 to 1996 (*n* = 385,180)

Gestational age	ANI (*n* = 253,716)			AI (*n* = 20,360)			FB-LIC (*n* = 1265)			FB-LMIC (*n* = 13,890)			FB-UMIC (*n* = 12,469)			FB-HIC (*n* = 83,480)		
	n	cRRR, (95%CI)	aRRR^a^ (95%CI)	n	cRRR, (95%CI)	aRRR^a^ (95%CI)	n	cRRR, (95%CI)	aRRR^a^ (95%CI)	n	cRRR, (95%CI)	aRRR^a^ (95%CI)	n	cRRR, (95%CI)	aRRR^a^ (95%CI)	n	cRRR, (95%CI)	aRRR^a^ (95%CI)
Extreme Preterm *(< 28 weeks)*	1403	Ref	Ref	301	3.37 (2.97, 3.81)	3.20 (2.78, 3.68)	11	1.63 (0.90, 2.96)	1.59 (0.87, 2.89)	76	1.12 (0.89, 1.41)	1.04 (0.82, 1.31)	68	1.04 (0.82, 1.33)	0.98 (0.77, 1.25)	453	0.99 (0.89, 1.11)	0.96 (0.86, 1.07)
Very early preterm *(28–32 weeks)*	1561	Ref	Ref	313	3.15 (2.78, 3.56)	2.67 (2.33, 3.06)	12	1.60 (0.90, 2.83)	1.63 (0.92, 2.89)	86	1.14 (0.92, 2.83)	1.11 (0.89, 1.38)	74	1.02 (0.81, 1.29)	1.01 (0.80, 1.27))	554	1.09 (0.89, 1.11)	1.09 (0.99, 1.20)
Early preterm *(32–33 weeks)*	1891	Ref	Ref	323	2.68 (2.38, 3.02)	2.52 (2.21, 2.88)	9	0.99 (0.51, 1.91)	0.98 (0.51, 1.89)	104	1.14 (0.92, 1.42)	1.09 (0.89, 1.33)	98	1.12 (0.92, 1.37)	1.08 (0.88, 1.33)	677	1.10 (1.01, 1.20)	1.08 (0.99, 1.18)
Late preterm *(34–36 weeks)*	11,263	Ref	Ref	2136	2.98 (2.83, 3.13)	2.80 (2.65, 2.95)	55	1.02 (0.77, 1.34)	1.00 (0.77, 1.33)	761	1.40 (1.30, 1.51)	1.35 (1.25, 1.46)	608	1.16 (1.07, 1.27)	1.13 (1.04, 1.23)	3970	1.09 (1.05, 1.13)	1.07 (1.03, 1.11)
Early term *(37–38 weeks)*	58,132	Ref	Ref	5727	1.55 (1.50, 1.60)	1.61 (1.55, 1.67)	316	1.13 (0.99, 1.29)	1.08 (0.95, 1.23)	4196	1.50 (1.44, 1.55)	1.42 (1.37, 1.48)	3296	1.22 (1.17, 1.27)	1.16 (1.11, 1.21)	19,597	1.04 (1.02, 1.06)	1.01 (0.99, 1.03)
Full term *(39–41 weeks)*	178,157			11,353			856			8594			8265			57,817		
Post term *(42 > weeks)*	1309	Ref	Ref	207	2.48 (2.14, 2.88)	1.58 (1.34, 1.85)	6	0.95 (0.43, 2.13)	1.11 (0.49, 2.47)	73	1.16 (0.91, 1.46)	1.29 (1.01, 1.64)	60	0.99 (0.76, 1.28)	1.12 (0.86, 1.45)	412	0.97 (0.87, 1.08)	1.05 (0.94, 1.17)

*ANI* Australian-born mothers of non-Indigenous backgrounds, *AI* Australian-born mothers of Indigenous background, *FB-LIC* foreign-born mothers from low-income countries, *FB-LMIC* foreign-born mothers from lower-middle-income countries, *FB-UMIC* foreign-born mothers of upper-middle-income countries, *FB-HIC* foreign-born mothers from high-income countries, *cRRR* crude relative risk ratio, *aRRR* adjusted relative risk ratio, *CI* confidence interval, *Ref* reference group

^a^ Adjusted for parity, birth year, SEIFA score, maternal age, and sex

**Table 3:** Risk of pre/post term relative to full term by maternal country of birth: 1997–2010 (*n* = 345,837)

Gestational age	ANI (*n* = 231,950)			AI (*n* = 20,206)			FB-LIC (*n* = 2840)			FB-LMIC (*n* = 17,951)			FB-UMIC (*n* = 17,452)			FB-HIC (*n* = 55,438)		
	n	cRRR, (95%CI)	aRRR^a^ (95%CI)	n	cRRR, (95%CI)	aRRR^a^ (95%CI)	n	cRRR, (95%CI)	aRRR^a^ (95%CI)	n	cRRR, (95%CI)	aRRR^a^ (95%CI)	n	cRRR, (95%CI)	aRRR^a^ (95%CI)	n	cRRR, (95%CI)	aRRR^a^ (95%CI)
Extreme preterm *(< 28 weeks)*	1457	Ref	Ref	370	3.27 (2.91, 3.67)	2.75 (2.41, 3.14)	30	1.46 (1.02, 2.11)	1.42 (0.98, 2.04)	137	1.29 (1.08, 1.54)	1.32 (1.10, 1.57)	106	1.00 (0.82, 1.22)	1.02 (0.83, 1.24)	349	0.99 (0.88, 1.12)	0.96 (0.85, 1.08)
Very early preterm *(28–32 weeks)*	1269	Ref	Ref	321	3.26 (2.88, 3.69)	2.73 (2.37, 3.14)	25	1.40 (0.94, 2.08)	1.37 (0.92, 2.04)	121	1.31 (1.08, 1.58))	1.34 (1.11, 1.62)	97	1.05 (0.85, 1.29)	1.08 (0.87, 1.33)	313	1.02 (0.90, 1.16)	0.99 (0.88, 1.13)
Early preterm *(32–33 weeks)*	1570	Ref	Ref	347	2.85 (2.53, 3.20)	2.59 (2.27, 2.96)	16	0.72 (0.44, 1.19)	0.71 (0.43, 1.17)	130	1.14 (0.95, 1.36)	1.16 (0.97, 1.39)	103	0.90 (0.74, 1.10)	0.91 (0.74, 1.11)	402	1.06 (0.95, 1.19)	1.02 (0.91, 1.14)
Late preterm *(34–36 weeks)*	11,822	Ref	Ref	1900	2.07 (1.96, 2.18)	1.91 (1.81, 2.03)	100	0.60 (0.49, 0.74)	0.60 (0.50, 0.74)	958	1.11 (1.04, 1.19)	1.14 (1.07, 1.23)	865	1.00 (0.94, 1.08)	1.01 (0.94, 1.09)	2659	0.93 (0.89, 0.97)	0.89 (0.85, 0.93)
Early term *(37–38 weeks)*	73,135	Ref	Ref	6173	1.09 (1.05, 1.12)	1.20 (1.17, 1.25)	656	0.64 (0.58, 0.70)	0.62 (0.57, 0.68)	6196	1.16 (1.12, 1.20)	1.15 (1.11, 1.19)	5903	1.11 (1.07, 1.15)	1.06 (1.02, 1.09)	17,311	0.98 (0.96, 1.00)	0.90 (0.88, 0.92)
Full term *(39–41 weeks)*	142,379			11,054			2004			10,386			10,360			34,345		
Post term *(42 > weeks)*	318	Ref	Ref	41	1.67 (1.20, 2.30)	1.73 (1.20, 2.48)	9	2.01 (1.03, 3.90)	1.93 (0.99, 3.78)	23	0.99 (0.65, 1.51)	0.98 (0.64, 1.50)	18	0.78 (0.48, 1.25)	0.76 (0.47, 1.22)	59	0.77 (0.58, 1.01)	0.73 (0.55, 0.97)

*ANI* Australian-born mothers of non-Indigenous backgrounds, *AI* Australian-born mothers of Indigenous background, *FB-LIC* foreign-born mothers from low-income countries, *FB-LMIC* foreign-born mothers from lower-middle-income countries, *FB-UMIC* foreign-born mothers of upper-middle-income countries, *FB-HIC* foreign-born mothers from high-income countries, *cRRR* crude relative risk ratio, *aRRR* adjusted relative risk ratio, *CI* confidence interval, *Ref* reference group

^a^ Adjusted for parity, birth year, SEIFA score, maternal smoking status, maternal age, and sex

**Table 4:** Risk of growth restriction compared to normal growth by maternal country of birth: 1980–1996 (*n* = 355,026)

POBW (%)	ANI (*n* = 234,195)			AI (*n* = 18,452)			FB-LIC (*n* = 1163)			FB-LMIC (*n* = 12,730)			FB-UMIC (*n* = 11,463)			FB-HIC (*n* = 77,023)		
	n	cRRR, (95%CI)	aRRR^a^ (95%CI)	n	cRRR, (95%CI)	aRRR^a^ (95%CI)	n	cRRR, (95%CI)	aRRR^a^ (95%CI)	n	cRRR, (95%CI)	aRRR^a^ (95%CI)	n	cRRR, (95%CI)	aRRR^a^ (95%CI)	n	cRRR, (95%CI)	aRRR^a^ (95%CI)
< 75 severe	6447	Ref	Ref	1222	2.92 (2.73, 3.12)	2.45 (2.27, 2.64)	30	0.96 (0.66, 1.39)	1.01 (0.69, 1.46)	375	1.09 (0.97, 1.21)	1.09 (0.98, 1.21)	290	0.92 (0.81, 1.04)	0.94 (0.83, 1.07)	2298	1.09 (1.04, 1.15)	1.11 (1.05, 1.16)
75–84 moderate	21,100	Ref	Ref	2642	1.93 (1.83, 2.02)	1.68 (1.59, 1.77)	109	1.06 (0.86, 1.31)	1.10 (0.89, 1.37)	1281	1.13 (1.06, 1.21)	1.15 (1.08, 1.23)	1010	0.98 (0.91, 1.05)	1.00 (0.94, 1.08)	7282	1.06 (1.02, 1.09)	1.07 (1.04, 1.11)
85–94 mild	60,105	Ref	Ref	5284	1.35 (1.30, 1.41)	1.27 (1.22, 1.32)	336	1.15 (0.99, 1.33)	1.17 (1.00, 1.36)	3446	1.07 (1.02, 1.12)	1.08 (1.03, 1.13)	2941	1.00 (0.95, 1.05)	1.02 (0.97, 1.07)	20,028	1.02 (1.00, 1.04)	1.03 (1.00, 1.05)
95–104	76,969			5002			375			4122			3765			25,172		
105–114	47,897	Ref	Ref	2663	0.86 (0.82, 0.90)	0.89 (0.84, 0.93)	199	0.85 (0.72, 1.01)	0.84 (0.71, 0.99)	2328	0.91 (0.86, 0.96)	0.88 (0.84, 0.94)	2298	0.98 (0.93, 1.03)	0.96 (0.92, 1.02)	15,192	0.97 (0.95, 0.99)	0.96 (0.94, 0.98)
115–124	15,936	Ref	Ref	982	0.95 (0.88, 1.02)	0.99 (0.92, 1.07)	87	1.12 (0.89, 1.42)	1.09 (0.86, 1.38)	810	0.95 (0.88, 1.03)	0.91 (0.84, 0.98)	849	1.09 (1.00, 1.18)	1.05 (0.97, 1.14)	5167	0.99 (0.96, 1.03)	0.97 (0.94, 1.00)
≥125	5741	Ref	Ref	657	1.76 (1.62, 1.92)	1.76 (1.60, 1.93)	27	0.97 (0.65, 1.43)	0.94 (0.63, 1.39)	368	1.20 (1.07, 1.34)	1.13 (1.00, 1.26)	310	1.10 (0.98, 1.24)	1.05 (0.94, 1.19)	1884	1.00 (0.95, 1.06)	0.97 (0.92, 1.03)

*ANI* Australian-born mothers of non-Indigenous backgrounds, *AI* Australian-born mothers of Indigenous background, *FB-LIC* foreign-born mothers from low-income countries, *FB-LMIC* foreign-born mothers from lower-middle-income countries, *FB-UMIC* foreign-born mothers of upper-middle-income countries, *FB-HIC* foreign-born mothers from high-income countries, *cRRR* crude relative risk ratio, *aRRR* adjusted relative risk ratio, *CI* confidence interval, *Ref* reference group

^a^ Adjusted for parity, birth year, SEIFA score, maternal age, and sex

**Table 5:** Risk of growth restriction compared to normal growth by maternal country of birth: 1997–2010 (*n* = 227,118)

POBW (%)	ANI (*n* = 151,295)			AI (*n* = 12,402)			FB-LIC (*n* = 2064)			FB-LMIC (*n* = 11,997)			FB-UMIC (*n* = 11,493)			FB-HIC (*n* = 37,867)		
	n	cRRR, (95%CI)	aRRR^a^ (95%CI)	n	cRRR, (95%CI)	aRRR^a^ (95%CI)	n	cRRR, (95%CI)	aRRR^a^ (95%CI)	n	cRRR, (95%CI)	aRRR^a^ (95%CI)	n	cRRR, (95%CI)	aRRR^a^ (95%CI)	n	cRRR, (95%CI)	aRRR^a^ (95%CI)
< 75 severe	3617	Ref	Ref	919	4.06 (3.75, 4.41)	2.64 (2.42, 2.89)	64	1.38 (1.07, 1.79)	1.69 1.30, 2.20)	371	1.34 (1.21, 1.51)	1.72 (1.53, 1.93)	260	0.96 (0.84, 1.09)	1.17 (1.03, 1.33)	907	0.98 (0.91, 1.06)	1.03 (0.95, 1.11)
75–84 moderate	11,611	Ref	Ref	2029	2.79 (2.63, 2.97)	1.99 (1.86, 2.12)	202	1.36 (1.16, 1.60)	1.54 (1.32, 1.81)	1178	1.33 (1.25, 1.43)	1.59 (1.48, 1.70)	964	1.10 (1.03, 1.19)	1.28 (1.19, 1.38)	3005	1.01 (0.97, 1.06)	1.06 (1.00, 1.10)
85–94 mild	36,115	Ref	Ref	3475	1.54 (1.46, 1.62)	1.34 (1.27, 1.40)	563	1.22 (1.09, 1.37)	1.28 (1.14, 1.43)	3353	1.22 (1.16, 1.28)	1.31 (1.25, 1.38)	2934	1.08 (1.03, 1.14)	1.14 (1.09, 1.20)	9185	1.00 (0.97, 1.03)	1.01 (0.98, 1.04)
95–104	50,309			3146			643			3827			3782			12,829		
105–114	33,026	Ref	Ref	1753	0.85 (0.80, 0.90)	0.91 (0.85, 0.97)	407	0.96 (0.85, 1.09)	0.92 (0.82, 1.05)	2193	0.87 (0.83, 0.92)	0.84 (0.80, 0.89)	2387	0.96 (0.91, 1.01)	0.93 (0.88, 0.98)	7942	0.94 (0.91, 0.97)	0.93 (0.90, 0.96)
115–124	11,839	Ref	Ref	632	0.85 (0.78, 0.93)	0.95 (0.87, 1.05)	125	0.83 (0.68, 1.00)	0.76 (0.62, 0.92)	733	0.81 (0.75, 0.88)	0.76 (0.70, 0.83)	806	0.91 (0.84, 0.98)	0.86 (0.80, 0.93)	2887	0.96 (0.92, 0.97)	0.94 (0.90, 0.98)
≥125	4778	Ref	Ref	448	1.50 (1.35, 1.66)	1.70 (1.52, 1.90)	60	0.98 (0.75, 1.28)	0.90 (0.69, 1.18)	342	0.94 (0.84, 1.06)	0.87 (0.78, 0.98)	360	1.00 (0.90, 1.12)	0.95 (0.85, 1.06)	1112	0.91 (0.85, 0.98)	0.89 (0.83, 0.95)

*ANI* Australian-born mothers of non-Indigenous backgrounds, *AI* Australian-born mothers of Indigenous background, *FB-LIC* foreign-born mothers from low-income countries, *FB-LMIC* foreign-born mothers from lower-middle-income countries, *FB-UMIC* foreign-born mothers of upper-middle-income countries, *FB-HIC* foreign-born mothers from high-income countries, *cRRR* crude relative risk ratio, *aRRR* adjusted relative risk ratio, *CI* confidence interval, *Ref* reference group

^a^ Adjusted for parity, birth year, SEIFA score, maternal smoking status, maternal age, and sex

**Table 6:** Risk of critically low and fairly low Apgar scores compared to normal Apgar score by maternal country of birth: 1980–1996 (*n* = 396,636)

Apgar 5mins	ANI (*n* = 261,380)			AI (*n* = 21,125)			FB-LIC (*n* = 1293)			FB-LMIC (*n* = 14,185)			FB-UMIC (*n* = 12,753)			FB-HIC (*n* = 85,900)		
	n	cRRR, (95%CI)	aRRR^a^ (95%CI)	n	cRRR, (95%CI)	aRRR^a^ (95%CI)	n	cRRR, (95%CI)	aRRR^a^ (95%CI)	n	cRRR, (95%CI)	aRRR^a^ (95%CI)	n	cRRR, (95%CI)	aRRR^a^ (95%CI)	n	cRRR, (95%CI)	aRRR^a^ (95%CI)
Critically low	2517	Ref	Ref	447	2.25 (2.04, 2.49)	2.03 (1.82, 2.28)	17	1.37 (0.85, 2.21)	1.39 (0.86, 2.25)	134	0.98 (0.83, 1.17)	0.95 (0.80, 1.14)	121	0.98 (0.82, 1.18)	0.98 (0/81, 1.17)	828	1.00 (0.93, 1.08)	1.00 (0.92, 1.08)
Fairly low	3619	Ref	Ref	566	1.98 (1.81, 2.17)	1.67 (1.51, 1.84)	14	0.78 (0.46, 1.33)	0.85 (0.50, 1/45)	221	1.13 (0.98, 1.29)	1.16 (1.01, 1.33)	168	0.95 (0.81, 1.11)	1.01 (0.86, 1.18)	1219	1.03 (0.96, 1.10)	1.06 (1.00, 1.14)
Normal	255,244			20,112			1262			13,830			12,464			83,853		

*ANI* Australian-born mothers of non-Indigenous backgrounds, *AI* Australian-born mothers of Indigenous background, *FB-LIC* foreign-born mothers from low-income countries, *FB-LMIC* foreign-born mothers from lower-middle-income countries, *FB-UMIC* foreign-born mothers of upper-middle-income countries, *FB-HIC* foreign-born mothers from high-income countries, *cRRR* crude relative risk ratio, *aRRR* adjusted relative risk ratio, *CI* confidence interval, *Ref* reference group

^a^ Adjusted for parity, birth year, SEIFA score, maternal age, and sex

**Table 7:** Risk of critically low and fairly low Apgar scores compared to normal Apgar score, by maternal country of birth: 1997–2010 (*n* = 347,794)

Apgar 5mins	ANI (*n* = 233,250)			AI (*n* = 20,325)			FB-LIC (*n* = 2910)			FB-LMIC (*n* = 18,019)			FB-UMIC (*n* = 17,537)			FB-HIC (*n* = 55,753)		
	n	cRRR, (95%CI)	aRRR^a^ (95%CI)	n	cRRR, (95%CI)	aRRR^a^ (95%CI)	n	cRRR, (95%CI)	aRRR^a^ (95%CI)	n	cRRR, (95%CI)	aRRR^a^ (95%CI)	n	cRRR, (95%CI)	aRRR^a^ (95%CI)	n	cRRR, (95%CI)	aRRR^a^ (95%CI)
Critically low	1800	Ref	Ref	379	2.46 (2.20, 2.75)	2.03 (1.79, 2.31)	34	1.54 (1.09, 2.16)	1.52 (1.08, 2.15)	144	1.03 (0.87, 1.23)	1.07 (1.90, 1.27)	12,133	0.98 (0.82, 1.17)	1.02 (0.86, 1.22)	420	0.98 (0.88, 1.09)	0.98 (0.88, 1.09)
Fairly low	2162	Ref	Ref	350	1.89 (1.69, 2.12)	1.58 (1.391, 1.79)	55	2.07 (1.58, 2.71)	2.17 (1.65, 2.84)	158	0.95 (0.80, 1.11)	0.99 (0.83, 1.16)	123	0.75 (0.63, 0.91)	0.80 (0.67, 0.97)	520	1.01 (0.91, 1.11)	1.06 (0.96, 1.16)
Normal	233,250			19,596			2821			17,717			17,281			54,813		

*ANI* Australian-born mothers of non-Indigenous backgrounds, *AI* Australian-born mothers of Indigenous background, *FB-LIC* foreign-born mothers from low-income countries, *FB-LMIC* foreign-born mothers from lower-middle-income countries, *FB-UMIC* foreign-born mothers of upper-middle-income countries, *FB-HIC* foreign-born mothers from high-income countries, *cRRR* crude relative risk ratio, *aRRR* adjusted relative risk ratio, *CI* confidence interval, *Ref* reference group

^a^ Adjusted for parity, birth year, SEIFA score, maternal smoking status, maternal age, and sex

For the period from 1980 to 1996, children of FB-LIC mothers had a non-significantly increased risk of preterm birth compared to those of ANI mothers (extreme preterm adjusted relative risk ratio [aRRR] 1.59, 95% confidence interval [CI] 0.87, 2.89; very early preterm aRRR 1.63, 95% CI 0.92, 2.89). During this period children of FB-LMIC mothers had a small but significantly increased risk of preterm and post-term births (late preterm aRRR 1.35, 95% CI 1.25, 1.46; early term aRRR 1.42, 95% CI 1.37,1.48; post-term aRRR 1.29, 95% CI 1.01, 1.64). Children of AI mothers had significantly increased risk in all categories of preterm and post term births during this period. For the period from 1997 to 2010, a non-significant increased risk of preterm and post-term births was observed for FB-LIC mothers (extreme preterm aRRR 1.42, 95% CI 0.98, 2.04; very early preterm aRRR 1.37, 95% CI 0.92, 2.04; post-term aRRR 1.93, 95 CI 0.99, 3.78). FB-LMIC mothers also had increased risks of extreme preterm (aRRR 1.32, 95% CI 1.10, 1.57) and very early preterm (aRRR 1.34, 95% CI 1.11, 1.62) births. Whilst a slight increased risk of late- preterm (aRRR 1.13, 95% CI 1.04, 1.23) and early term births (aRRR 1.16, 95% CI 1.11, 1.21) was seen in children of FB-UMIC during the first period and an only slightly increased risk of early term in the second period, children of FB-HIC had only a very slight increased risk of late preterm birth during the first period (aRRR 1.07, 95% CI 1.03, 1.11).

Children of AI mothers had increased risk of severe IUGR and increased risk of excessive fetal growth during the period from 1980 to 1996 (Table 4). There was a slight increase in risk of mild growth restriction for children of FB-LIC mothers (aRRR 1.17, 95% CI 1.00, 1.36), and also a slightly elevated risk of mild (aRRR 1.08, 95% CI 1.03, 1.13) and moderate IUGR (aRRR 1.15, 95% CI 1.08, 1.23) for children of FB-LMIC mothers. IUGR was more evident in children of AI and in those of foreign-born women from low and lower middle-income countries during the second period (1997–2010) of this study (Table 5). During this period for children of FB-LIC and FB-LMIC mothers, there were increases in severe (aRRR 1.69, 95% CI 1.30, 2.20; aRRR 1.72, 95% CI 1.53, 1.93), moderate (aRRR 1.54, 95% CI 1.32, 1.81; aRRR 1.59, 95% CI 1.48, 1.70) and mild (aRRR 1.28, 95% CI 1.14, 1.43; aRRR 1.31, 95% CI 1.25, 1.38) IUGR compared to children of ANI mothers. Children of FB-UMIC mothers had increased risk of severe (aRRR 1.17, 95% CI 1.03,1.33), moderate (aRRR 1.28, 95% CI 1.19,1.38) and mild (aRRR 1.14, 95% CI 1.09,1.20) IUGR during the second period only (Table 5), whilst children of FB-HIC mothers had very slight increased risks of severe, moderate and mild IUGR during the first periods (Table 4).

Compared to children of ANI mothers, children of FB-LIC mothers had an increased adjusted relative risk of critically low 5-min Apgar score (1997–2000 aRRR 1.52, 95% CI 1.08, 2.15) and an elevated risk of fairly low 5-min Apgar scores in the second period (aRRR 2.17, 95% CI 1.65, 2.85) (Table 7). Again, increased risks were observed in critically low and fairly low Apgar scores at 5 min for children of AI mothers. However, FB-HIC mothers had a minimally increased risk of fairly low Apgar score in the first time period (Table 6).

## Discussion

The proportion of foreign born women in our dataset was consistent with current data from the Australian Bureau of Statistics reporting 28% of Australians are born overseas [33]. However we identified considerable variation in the distribution of demographic characteristics across our study groups. A higher proportion (27%) of Australian-born Indigenous mothers were less than 20 years of age), while Australian-born non-Indigenous and foreign-born mothers were more likely to be married (90.1 to 93.2% compared 62.8% of Australian-born Indigenous mothers). Over a third of Australian-born Indigenous mothers were in the most socio-economically disadvantaged category as were 13.3% of FB-LMIC and 12.9% of FB-LIC mothers compared to only 9.9% of ANI mothers, whilst smoking during pregnancy was most prevalent in Australian-born Indigenous mothers and least prevalent in foreign-born mothers. Although foreign-born women from countries of low and middle income accounted for 9% of the Western Australian childbearing population and these mothers were more likely to be older and married, their infants may have been at some increased risk of extreme prematurity (< 28 weeks), very early preterm birth (28–32 weeks), late preterm birth (34–36 weeks), early term (37–38 weeks) and post term (42 > weeks), as well as IUGR (severe, moderate and mild) for one or both of the time periods. However it is important to be cautious about the strength of this evidence particularly in relation to preterm birth given the extremely small sample sizes in some of these groups and the associated wide confidence intervals. Furthermore, children of foreign-born low income mothers also did have an increased risks of both critically low and fairly low 5-min Apgar scores and the evidence for this was stronger than for some of the preterm birth categories. However these poorer perinatal and neonatal outcomes were not seen in children of foreign-born mothers from high-income countries, whose better outcomes mirrored our comparative group (children of Australian-born non-Indigenous mothers). Preterm birth rates have increased worldwide [3] but in WA the rate has been relatively stable between 1980 and 2010 and with an average of 7.2% children each year born prematurely (Fig. 1), whilst post term births decreased over time (Fig. 2).

Our findings of an increased risk of adverse infant outcomes for Indigenous mothers are consistent with our previous analyses [36] and previous literature which has found that IUGR [37, 38] and low birth weight [39] are more prevalent in this population. The underlying causes for these disparities are believed to be multiple, extending from specific risk factors, such as maternal smoking and medical conditions, to more complex societal issues related to social disadvantage [40].

The strengths of this study include the use of population-based data over a 30-year period from the WA Data linkage system. This infrastructure has been very effective in linking data from national and local health and welfare data sets and has been a cost effective way of improving health policy and practice [41]. Another strength is the use of POBW, which is superior to weight z scores and percentiles (which only compare a given birthweight with the average birthweight for all sex-specific births at the same gestational age) because it relates to the average birthweight for live births with the same sex, maternal age, maternal height, and maternal parity, and without any known risk factors (such as smoking) for pre-term birth or adverse birth outcomes [34, 35]. Moreover, the model from which it has been derived has also been validated against fetal weights estimated from serial biometric ultrasound scans at least for infants over 29 weeks’ gestation [42].

In our dataset we were unable to adjust for maternal medical conditions and pregnancy complications, as the data were incomplete for these variables. Furthermore, the population sources do not include detailed information on maternal education and other important maternal variables such as weight, nutrition, lived trauma and adverse child experiences, which could have provided a fuller picture. As such, our data were limited to maternal country of birth as the only indicator of immigration status. Further data such as age of arrival into Australia and immigration status would also have been valuable and remains a limitation in immigrant and refugee identification for this study [43].

An increased risk of adverse pregnancy and birth outcomes among foreign-born women compared to native-born women in our study has also been demonstrated in several other studies [11, 13, 16, 18, 19, 44]. A 2012 Danish study found that foreign-born women had increased risk of having a baby small for gestational age, with the highest risk observed among mothers from Lebanon, Somalia, and Pakistan [16] countries which we would have classified as ‘low and middle income’. In the same study children of foreign-born mothers were more likely to be born very preterm (< 33 weeks) or moderately preterm (33–36 weeks) [16]. In contrast to our study, these data included detailed immigration information such as length of residence in Denmark, age at immigration and immigration status but relationships between these variables and the child’s birth outcomes were not found [16]. A Canadian study reported higher rates of caesarean sections in women from Latin America, the Caribbean and Sub-Saharan Africa compared to Canadian-born women, but in contrast to our study, they showed a lower risk of preterm delivery, findings the authors postulated might be associated with a healthy migrant effect [13]. However, this study also reported a three-fold increase risk of low birth weight compared with those of Canadian-born women. We elected to investigate IUGR rather than birth weight because IUGR is a better predictor of poorer birth outcomes as it refers to deviation and reduction in the expected fetal growth during pregnancy [5]. A recent US study also found that foreign-born non-Hispanic black women had significantly lower rates of preterm birth and small for gestational age in comparison to US-born non-Hispanic black women [15]. There were also significantly lower rates of preterm birth and intra-uterine growth restriction among black women from Sub-Saharan Africa compared to native Caribbean-born black women [15]. Authors of a Swedish cohort study suggested that an increase in preterm birth in foreign-born women could be the result of the stress experienced during migration and the fact that female refugees of child-bearing age are more likely to be victims of trauma, traumatic events, acts of violence and thus more vulnerable to stress exposure [44].

An Australian study investigating how antenatal and perinatal characteristics compared between children diagnosed with ID, those diagnosed with ASD and those diagnosed with ASD and ID, found that any category of preterm birth was associated with a two-fold increased risk of mild and severe ID [8]. Another data linkage study with clinical cases linked to midwives data in New South Wales, Australia, found that having a mother born outside of Australia was one of four risk factors which increased the likelihood of a subsequent autism diagnosis [45]. Our findings of prematurity and foreign-born mothers, shows that children of immigrant backgrounds are at an increased risk of the subsequent development of neurodevelopmental disabilities such as ASD, as previously found [45].

IUGR was an outcome in our study associated consistently across the two time periods with an increased risk for infants born to foreign women from low to middle income countries. Its presence has also been linked to the subsequent development of neurodevelopmental disabilities [36]. Poor fetal growth can be determined by measuring POBW and, when compromised, has been shown in one WA study to be strongly associated with an increased risk of mild to moderate ID, severe ID and ASD associated with ID but not ASD without ID [8]. An earlier WA study also found that severe IUGR (POBW < 75) was associated with a threefold increased risk in severe ID [36]. Also, a child with POBW of less than 85% was two to three times more likely to be diagnosed with mild to moderate ID compared to a child with POBW of 95 to 104% (normal POBW), and this likelihood increased to more than four times for severe ID [8]. Severe IUGR was associated with four times the risk for severe ID, and also more than three times the risk for ASD with ID [8]. These associations of IUGR with ASD and ID are concerning because of our findings that children of foreign-born women from low and middle-income countries are at increased risk of IUGR and hence of such neurodevelopmental disabilities and disorders.

The American Academy of Paediatrics states that in predicting outcomes, a 5 min Apgar score of 0 to 3 correlates with neonatal mortality in large populations but does not predict future neurologic dysfunction [7]. In contrast a low 5 min Apgar score has been shown to increase the risk of cerebral palsy, with 20 to 100 times the risk compared to 5 min Apgar scores from 7 to 10 [7]. A Scottish study found that a low Apgar score at 5 min was strongly associated with the risk of neonatal and infant death [6]. Our study found children of foreign-born women from low and middle-income countries had an increased risk of critically low (0 to 3) and fairly low (4 to 6) Apgar scores, particularly after adjustment for birth year, gender, maternal age, SEIFA score, parity and smoking.

Our findings illustrate the vulnerabilities of children born to foreign women from low and middle-income countries. Our findings show that for some time periods pregnancy and birth outcomes can be influenced by maternal country of birth, especially if mothers come from low and middle-income countries where they could have been exposed to factors such as war, trauma, poverty, and limited opportunities for education and lack of access to proper healthcare. The higher perinatal risk observed in the second period of this study, 1997 to 2010 perhaps demonstrates the changing dynamics of foreign-born populations arriving in Australia from low and middle-income countries. Clinically these results illustrate the need for ongoing monitoring and targeting of foreign-born women during their antenatal care to reduce risks of adverse pregnancy and birth outcomes. A US study suggested that the patterns of prematurity, mortality, and small for gestational age experienced by infants of immigrant women, can lead to an increased risk of long term medical, developmental, and economic disadvantage for children and their families [15].

## Conclusion

This study determined that children of foreign-born women from low and middle-income countries had a greater risk of preterm and post term birth, intra uterine growth restriction and lower Apgar scores compared to children of Australian-born non-Indigenous mothers. These results indicate the need for strategies that could be implemented to address immigrant health, particularly targeting antenatal care in order to reduce risks of poor pregnancy, neonatal, and birth outcomes. Policy makers also need to implement more culturally sensitive strategies to provide services to these groups of families. Highlighting and addressing multicultural issues could improve these outcomes and the cultural needs of immigrant women by medical and allied health care providers.

## Additional files

Additional file 1: **Table S1.** Low and middle-income countries. (PDF 251 kb)

Additional file 2: **Table S2.** High income countries. (PDF 243 kb)

## Data Availability

Our ethical approval does not allow for sharing of unpublished data. If such data were required an application would need to be made to the WA Department of Health Human Research Ethics Committee.

## Abbreviations

AI: Australian-born mothers of indigenous background (Aboriginal and Torres Strait Islander)
ANI: Australian-born mothers of non-indigenous backgrounds (Caucasian)
ASD: Autism Spectrum Disorders
CALD: Culturally and linguistically diverse backgrounds
CP: Cerebral Palsy
FB-HIC: Foreign-born mothers from high income countries
FB-LIC: Foreign-born mothers from low income countries
FB-LMIC: Foreign-born mothers from lower middle-income countries
FB-UMIC: Foreign-born mothers from upper middle-income countries
ID: Intellectual Disability
IRSAD: Index of Relative Socio-economic Advantage and Disadvantage
IUGR: Intra uterine growth restriction
MCB: Maternal country of birth
POBW: Proportion of optimal birth weight
SEIFA: Socio-Economic Indexes for Areas
WA: Western Australian
