# Supplementary material for: Are preterm birth and intra-uterine growth restriction more common in Western Australian children of immigrant backgrounds? A population based data linkage study
Source: BMC Pregnancy Childbirth. 2019 Aug 9;19:287. doi: 10.1186/s12884-019-2437-x (PMC6688266; doi:10.1186/s12884-019-2437-x)
Supplement: Supplementary file 1 — Table S1. Low and middle-income countries. (PDF 251 kb) [file 12884_2019_2437_MOESM1_ESM.pdf]

Supplementary Table S1: Low and middle-income countries

| Low Income countries | | Lower Middle-Income countries | | Upper Middle-Income countries | |
|---|---|---|---|---|---|
| Afghanistan | Malawi | Angola | Mauritania | Albania | Maldives |
| Bhutan | Mali | Armenia | Moldova | Algeria | Mauritius |
| Bolivia | Mozambique | Bangladesh | Mongolia | Argentina | Mexico |
| Borneo | Nepal | Burma | Morocco | Belarus | Namibia |
| Burkina Faso | Palestine | Cambodia | Nicaragua | Bosnia | Nauru |
| Burundi | Rwanda | Cameroon | Nigeria | Botswana | Panama City |
| Central African Rep | Senegal | Cape Verde | Pakistan | Brazil | Paraguay |
| Chad | Sierra Leone | Colombia | Papua New Guinea | Bulgaria | Peru |
| Congo | Somalia | Djibouti | Philippines | China | Romania |
| Eritrea | Tanzania | Egypt | Solomon Islands | Costa Rica | Rumania |
| Ethiopia | Togo | El Salvador | Sri Lanka | Croatia | Russia |
| Gambia | Uganda | Ghana | Sudan | Cuba | Samoa |
| Guinea | | Guatemala | Swaziland | Dominican Republic | Serbia |
| Liberia | | Honduras | Syria | Ecuador | South Africa |
| Madagascar | | India | Tajikistan | Falkland Islands | Suriname |
| | | Indonesia | Timor | Fiji | Tahiti |
| | | Ivory Coast | Tunisia | Guyana | Thailand |
| | | Jordan | Ukraine | Iran | Tonga |
| | | Kenya | Uzbekistan | Iraq | Turkey |
| | | Kiribati | Vanuatu | Kazakhstan | Tuvalu |
| | | Kyrgyzstan | Vietnam | Lebanon | Venezuela |
| | | Laos | Yemen | Libya | West Indies |
| | | Lesotho | Zambia | Malaysia | Yugoslavia |
