# Supplementary material for: Are preterm birth and intra-uterine growth restriction more common in Western Australian children of immigrant backgrounds? A population based data linkage study
Source: BMC Pregnancy Childbirth. 2019 Aug 9;19:287. doi: 10.1186/s12884-019-2437-x (PMC6688266; doi:10.1186/s12884-019-2437-x)
Supplement: Supplementary file 2 — Table S2. High income countries. (PDF 243 kb) [file 12884_2019_2437_MOESM2_ESM.pdf]

Supplementary Table S2: High income countries

High Income countries					
Australia	Austria	Bahamas	Bahrain	Belgium	Bermuda
Canada	Channel Islands	Chile	Cook Islands	Cyprus	Czech Republic
Denmark	Diego Garcia	Dutch West Indies	England	Estonia	Finland
France	French Polynesia	Germany	Gibraltar	Greece	Greenland
Guadeloupe	Guam	Hong Kong	Hungary	Iceland	Ireland
Isle of Man	Israel	Italy	Japan	Korea	Kuwait
Latvia	Liechtenstein	Lithuania	Luxembourg	Malta	Netherlands
New Caledonia	New Hebrides	New Zealand	Niue Island	Norway	Oman
Poland	Portugal	Puerto Rico	Qatar	Reunion Island	Saudi Arabia
Scotland	Seychelles	Singapore	Slovakia	Slovenia	Spain
Sweden	Switzerland	Taiwan	UK	United Arab Emirates	Uruguay
USA	Virgin Islands	Wales			
